# Semaphorin 7A Promotes VEGFA/VEGFR2-Mediated Angiogenesis and Intraplaque Neovascularization in *ApoE^-/-^* Mice

**DOI:** 10.3389/fphys.2018.01718

**Published:** 2018-11-30

**Authors:** Shuhong Hu, Yifei Liu, Tao You, Li Zhu

**Affiliations:** Cyrus Tang Hematology Center, Collaborative Innovation Center of Hematology of Jiangsu Province, State Key Laboratory of Radiation Medicine and Protection, Soochow University, Suzhou, China

**Keywords:** semaphorin 7A, integrin β1, VEGFA/VEGFR2, angiogenesis, neovascularization

## Abstract

Excessive neovascularization of atherosclerotic lesions increases plaque vulnerability and the susceptibility to rupture. Semaphorin 7A (Sema7A), a semaphorin family member, was recently reported to promote atherosclerotic plaque formation by mediating d-flow-induced endothelial phenotypic change and leukocyte adhesion. To extend our understanding of the proatherogenic role of Sema7A, we investigated the role of endothelial Sema7A in angiogenesis and atherosclerotic neovascularization. Sema7A overexpression in human umbilical vein endothelial cells (HUVECs) significantly upregulated VEGFA/VEGFR2 and promoted cell migration and angiogenesis. This enhancing effect was eliminated by the blockage of Sema7A receptor, β1 integrin. Inhibition of FAK or ERK1/2 downstream of β1 integrin signaling significantly inhibited cell migration and angiogenesis via ROCK (Rho-associated coiled forming protein kinase) and MYPT (myosin phosphatase targeting subunit), which are responsible for actin polymerization. Consistently, *in vivo* studies showed a remarkable reduction in VEGFA/VEGFR2 expression and neovascularization in the atherosclerotic plaques of *Sema7A^-/-^ApoE^-/-^* mice compared with *Sema7A^+/+^ApoE^-/-^* littermates. Supportively, Sema7A deficiency reduced the accumulation of T cells, macrophages, and dendritic cells, and enhanced plaque stability in *ApoE^-/-^* mice. Together, our findings show that Sema7A promotes VEGFA/VEGFR2-mediated neovascularization in a β1 integrin-dependent manner, supporting a crucial role of Sema7A in the progression of human atherosclerosis.

## Introduction

The major cause of acute cardiovascular events is the rupture or erosion of an atherosclerotic plaque (Nus and Mallat, 2016). Plaque angiogenesis and neovascularization, including the subsequent intraplaque hemorrhage, contribute to plaque growth and instability (Parma et al., 2017). Generally, normal vessel walls contain a microvasculature located at the adventitia, which is called the adventitial vasa vasorum (Barger et al., 1984). The vasa vasorum normally presents as an organized network of vessels paralleling to the artery and running through the vessel wall at regular intervals and bifurcates around the vessels (Kwon et al., 1998). However, around atherosclerotic plaque area, vasa vasorum covers more extensive area and penetrates into the intima of the lesion (Barger et al., 1984). Newly formed vessels fuel plaque growth and exacerbate plaque vulnerability at the earlier and later stages of atherosclerosis, respectively (Parma et al., 2017). Initially, intraplaque angiogenesis provides oxygen and nourishment to maintain plaque growth. However, sustained tissue damage with accompanying inflammation activates endothelial cells to increase vascular permeability and to promote the recruitment of inflammatory leukocytes (Bruno et al., 2014; Marra and Tacke, 2014; Novo et al., 2014). These cells produce and secrete pro-angiogenic cytokines and chemokines, leading to endothelial cell proliferation and migration, which are fundamental for angiogenesis and atherosclerosis in return (Simpson et al., 2003; Bosisio et al., 2014; Marra and Tacke, 2014; Tecchio and Cassatella, 2014), from the initial stages of atherosclerosis to acute cardiovascular events (Sedding et al., 2018). Therefore, exploring the underlying mechanism of intraplaque angiogenesis is crucial for the prevention and treatment of atherosclerosis.

Semaphorins belong to a large family of trans-membrane (including GPI-anchored) or secreted proteins, which were initially demonstrated to mediate neuron-axonal guidance, are recently reported to be involved in multiple diseases. In the process of angiogenesis, the targets of semaphorins are normally the cytoskeleton proteins and focal adhesions. Focal adhesion molecules are dynamic cell-extracellular matrix adhesive structures upon integrin binding. Semaphorin signaling affects the assembly/disassembly of focal adhesions and induces cytoskeleton reorganization, resulting in changes of cell shape, vitality, and migration (Serini et al., 2008; Gelfand et al., 2009).

Semaphorin 7A (Sema7A) is the only class 7 semaphorin member with an GPI anchored domain. The Sema7A molecule consists of a Sema domain, an N-terminal seven-bladed β converted propeller, a plexin-semaphorin-integrin domain, an immunoglobulin-like domain, and an GPI anchor in C-terminal domain. Studies showed that Sema7A mediates atherosclerosis (Hu et al., 2018), retinal angiogenesis (Ghanem et al., 2011), and tumor growth (Garcia-Areas et al., 2014). It is well known that the proliferation and migration of endothelial cells are fundamental for the atherosclerosis of angiogenesis and development. However, the role of Sema7A in angiogenesis during the development of atherosclerosis remains unclear. In this study, we demonstrated that genetic deletion of Sema7A ameliorates atherosclerotic neovascularization and plaque vulnerability in mice. Mechanistically, the pro-angiogenic function of Sema7A is mediated through β1 integrin and the activation of VEGFA/VEGFR2 axis. Using Sema7A-overexpressing HUVECs, we further showed that Sema7A promotes endothelial cell migration and angiogenesis through FAK/MAPK signaling pathway in a β1 integrin-dependent manner.

## Materials and Methods

### Mice

Animal researches were approved by the Institutional Animal Care and Use Committee of Soochow University. *Sema7A^-/-^, ApoE^-/-^* and WT mice with C57BL/6J background were obtained from the Jackson Laboratories (Bar Harbor, United States). *Sema7A^-/-^* and *ApoE^-/-^* mice were backcrossed on C57BL/6 for >10 generations. Investigators who performed the experiments were blinded to mouse genotypes.

### Analysis of Atherosclerotic Lesions

*Sema7A^+/+^ApoE^-/-^* and *Sema7A^-/-^ApoE^-/-^* mice were generated as previously described (Hu et al., 2018). The mice were fed a normal chow diet for 8 weeks before changing into a high-fat diet (HFD) (0.15% cholesterol and 21% fat without added cholate, Harlan Teklad, 88137, United States) for another 12 weeks (Saederup et al., 2008; Zhu et al., 2009).

### Immunostaining

For the aortic root analysis, mouse hearts were embedded in TissueTek O.C.T (4583, SAKURA, United States) and sections from the aortic root of 8-μm were fixed on slides. Sema7A was detected with a rabbit anti-mouse Sema7A antibody (ab23578, Abcam, United States). Endothelial cells were stained with a rat anti-mouse CD31 antibody (553370, BD Biosciences, United States). Monocytes and macrophages were detected with a rat anti-mouse MOMA-2 antibody (ab33451, Abcam, United States). T cells were stained with a rat anti-mouse CD4 antibody (ab25475, Abcam, United States). Dendritic cells (DCs) were detected with a hamster anti-mouse CD11c antibody (ab33483, Abcam, United States). A rabbit anti-mouse α-SMA antibody (Bs-0189, Bioss, China) was used for staining smooth muscle cells (SMCs). Fluorescence-labeled secondary antibodies (Alexa Fluor donkey anti-rabbit 488, Alexa Fluor rabbit-anti-mouse 555, Alexa Fluor donkey-anti-mouse 647, Abcam, United States) and Cy3 donkey anti-goat IgG (H+L) (A0502, Beyotime, China) were used according to the manufacturers’ protocols. Sections were counterstained with DAPI (C1002, Beyotime, China). Images were examined using a multicolor digital camera on an IX-81 laser confocal microscope (Olympus, Japan). MOMA-2-, CD4-, CD11c- and α-SMA-positive areas were obtained by image analysis (Olympus cellSens software). A neutrophil-staining kit (Sigma-Aldrich, 91C-KT, Germany) was used to identify neutrophils. Collagen was stained with Masson’s Trichrome (SBJ, China). Images were captured using a Leica DM2000 microscope (Germany) and Olympus camera (Japan) and analyzed by Olympus cellSens software. The results were shown as percent of the positive area in total plaque area.

### HUVEC Culture

Human umbilical vein endothelial cells (HUVECs) (ATCC, Manassas) were maintained in DMEM-low glucose medium with 10% FBS at 37°C with 5% CO_2_. Cultures were then starved with serum-free medium for 12 h before assays. All assays were conducted using 2–5 passage cells. HUVECs expressing hSema7A-pCDH-GFP or pCDH-GFP were transduced as previously (Hu et al., 2018).

### RNA Isolation and QPCR

Total RNAs from HUVECs were extracted using the QIAGEN miRNeasy Mini kit (217004, Qiagen, Germany). Isolated RNAs were reverse transcribed into cDNAs using the Takara PrimeScript^TM^ RT Master Mix (RR036A, Takara, Japan). QPCR was done in triplicates in 10 μl of the brilliant SYBR green PCR master mixture (4913914, Roche, Switzerland) in a real-time-PCR System (LightCycler 480, Roche, Switzerland). The mRNA levels were normalized to the glyceraldehyde-3-phosphate dehydrogenase (GAPDH) level and displayed as relative fold changes by the 2^-ΔΔCT^ methods. Sequences for the QPCR primers are listed in Supplementary Table S1.

### Western Blotting Analysis

The cells were washed with PBS and dissolved in RIPA buffer (1% Triton X-100, 1% deoxycholate, 0.1% SDS, 10 mM Tris and 150 mM NaCl) on the ice for 30 min, with protease and phosphatase inhibitor cocktail (78440, Thermo Fisher, United States) adding into the buffer. After lysis, cell lysates were centrifuged at 14000 *g* for 5 min at 4°C and abandoned the precipitation. Protein concentrations in the supernatants were measured by the Enhanced BCA Protein Assay Kit (P0010, Beyotime, China). Twenty μg proteins were heated at 95°C for 5 min following sample buffer (161-0737, Bio-Rad, United States) addition, and separated in 8% SDS-PAGE gels. Western blots were incubated with 5% non-fat milk (ERMBD150, Sigma-Aldrich, United States), washed with TBST, and probed with primary antibodies as follows: rabbit anti-human p-FAK (8556, Cell Signaling Technology, United States), rabbit anti-human FAK (3285, Cell Signaling Technology, United States), rabbit anti-human p-ERK1/2 (4376, Cell Signaling Technology, United States), rabbit anti-human ERK1/2 (9102, Cell Signaling Technology, United States), rabbit anti-human ROCK1 (3033, Cell Signaling Technology, United States), and rabbit anti-human MYPT1 (8574, Cell Signaling Technology, United States). After incubation at 4°C overnight and TBST washing, membranes were incubated with fluorescent secondary antibodies (goat anti-rabbit IRDye 800CW, goat anti-mouse IRDye 800CW, Licor Odyssey, United States). Membranes were examined by Odyssey infrared imaging system (LI-COR Biosciences, United States). Densitometry analysis was done using the ImageJ software (NIH) to quantify protein expression levels with β-actin expression level as a reference.

### Scratch Wound Assay

Human umbilical vein endothelial cells expressing hSema7A-pCDH-GFP or pCDH-GFP were seeded in 24-well plates and allowed to attach for 24 h. When HUVEC monolayer cultures reached approximately 95–100% confluence, the cells were pre-treated with blocking antibody or inhibitors (blocking antibodies for β1 (P5D2, ab24693, Abcam, United States), inhibitors for VEGFR2 (ZM323881, HY-15467, MCE, United States), FAK (PF573228, S2013, Selleck Chemicals, United States) and MAPK (U0126, 662005, Merck Millipore, Germany). Cells were starved with serum-free medium for 12 h before a scratch wound assay was carried out, as previously described (Chanakira et al., 2015). Briefly, three scratches were made across the wells by a 200-μl pipette tip. After plates were washed with PBS, HUVECs were grown for 24 h and images were captured at 0, 6, 24 h after preparing scratch wounds using a Nikon Microscope. The pictures were captured at the same magnification and size. Closure of the wound area was quantitated by Image-Pro Plus software. The total area of the blank region was measured and the average distance was obtained by dividing the total area by the height. Data are summarized as means ± SEM.

### Transwell Assay

Human umbilical vein endothelial cells expressing hSema7A- pCDH-GFP or pCDH-GFP (50,000) were pretreated same as Scratch wound assay and plated in the upper chamber of the BD BioCoat chamber (353097, BD Falcon, United States) with serum-free medium. And the migrated endothelial cells toward a gradient of 20% FBS in the lower chamber was monitored. After incubation for 24 h, removed the cells on the upper surface of the membrane; cells located on the lower surface of the membrane were fixed in 4% PFA for 20 min and then stained with crystal violet for 5 min. Numbers of stained cells were quantified by microscope-captured images.

### Tube Formation Assay on HUVEC *in vitro*

The effect of Sema7A on endothelial cells was detected by *in vitro* tube formation on Matrigel (356234, BD Biocoat, United States). Confluent Sema7A-pCDH-HUVECs and pCDH-HUVECs were harvested and diluted (12 × 10^4^ cells) in 500 μl serum-free medium, which were then plated on Matrigel-coated 24-well plates in triplicate at 37°C for 6 h. In details, cells pre-cultured with blocking antibody or inhibitors for 24 h before assay. Tube formation were examined under an inverted microscope after 6 h, images were captured with the Nikon AZ100M microscope. The number of tube and branching length were quantitated by the ImageJ Angiogenesis Analyzer plugin.

### Immunofluorescence and Laser Confocal Microscopy (LCMS)

Human umbilical vein endothelial cells expressing hSema7A-pCDH-GFP or pCDH-GFP were fixed in 4% PFA, permeabilized with 0.1% Triton X-100, washing with PBST, and co-stained with rhodamine phalloidin (PHDR1, Cytoskeleton, United States) or VE-Cadherin (AF1002-SP, R&D Systems, United States). Cell nuclei were stained with DAPI. Images were examined using a multicolor digital camera on an IX-81 laser confocal microscope (Olympus, Japan).

### Statistical Analysis

Data were analyzed by Prism 6.0 (GraphPad) software with unpaired two-tailed Student’s *t*-tests or one-way ANOVA followed by post-HOC analysis. Data were present as mean ± SEM (standard error of the mean). Differences were considered significant at *P <* 0.05.

## Results

### Deletion of Sema7A Reduces Intraplaque Neovascularization and VEGFA/VEGFR2 Expression in *ApoE^-/-^* Mice

**FIGURE 1:**
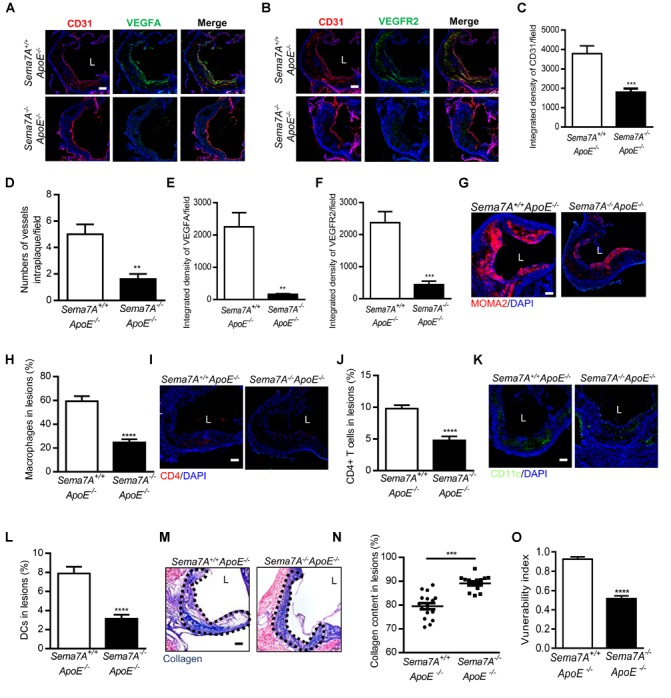
Sema7A deletion reduces intraplaque neovascularization via VEGFA/VEGFR2 downregulation and impairing inflammatory cell infiltration and remarkably enhances plaque stability in *ApoE^-/-^* mice. **(A,B)** Aortic root sections from *Sema7A^+/+^ApoE^-/-^* and *Sema7A^-/-^ApoE^-/-^* mice on HFD for 12 weeks. Endothelial cells were stained with CD31 and VEGFA/VEGFR2 (CD31, red; VEGFA/VEGFR2, green; nuclei, blue). Data are shown as mean ± SEM (n ≥ 10 mice per group). Bar = 100 μm. **(C,E,F)** Integrated density of CD31/VEGFA/VEGFR2 was qualified from **(A,B)**. Data are shown as mean ± SEM (*n* ≥ 10 mice per group). Bar = 100 μm, ^∗∗^*P* < 0.01; ^∗∗∗^*P* < 0.001. **(D)** Quantified data for numbers of vessels intraplaque/filed. Data are shown as mean ± SEM (*n* ≥ 10 mice per group). Bar = 100 μm, ^∗∗^*P* < 0.01. **(G–L)** Aortic root sections were also stained for macrophages (**G**, MOMA-2, red; nuclei, blue), T cells (**I**, CD4, red; nuclei, blue) and DCs (**K**, CD11c, green; nuclei, blue), Data are shown as mean ± SEM (*n* ≥ 10 mice per group). Bar = 100 μm. ^∗∗∗∗^*P* < 0.0001. **(M)** Collagen content (blue) in aortic root sections was stained with Masson’s Trichrome and nucleus was counterstained with hematoxylin. L, aortic lumen. **(N)** The result was shown as percent of the positive area in plaque area. Data are shown as mean ± SEM. *n* ≥ 10 mice per group. Bar = 200 μm. ^∗∗∗^*P* < 0.001. **(O)** The vulnerability index, an indicator of plaque stability, in *Seme7A^+/+^ApoE^-/-^* and *Seme7A^+/+^ApoE^-/-^* was calculated. Vulnerability index = (macrophage content % + lipid content %)/(collagen content % + VSMC content %) Data are shown as mean ± SEM, *n* ≥ 10, ^∗∗∗∗^*P* < 0.0001.

We recently demonstrated that Sema7A mediates d-flow-induced endothelial dysfunction and promotes atherosclerosis, and deletion of Sema7A significant reduces atherosclerotic plaque formation (Hu et al., 2018). Given a pro-angiogenic role of Sema7A in tumor and corneal cells (Ghanem et al., 2011; Garcia-Areas et al., 2014), we asked whether it mediates neovascularization in atherosclerosis. In atherosclerotic lesions, vascular endothelial growth factor (VEGF) is one of the crucial growth factors involved in angiogenesis (Weis and Cheresh, 2005). Expressed by endothelial cells, SMCs (smooth muscle cells), and macrophages (Di Stefano et al., 2009), VEGF functions as important endothelial cell regulator in cell proliferation, migration, and permeability modulation through its receptors (VEGFR) (Ferrara and Davis-Smyth, 1997). Therefore, we asked whether Sema7A is involved in intraplaque neovascularization and regulates angiogenesis through VEGFA/VEGFR2 axis in *Sema7A^+/+^ApoE^-/-^* mice on HFD. Intraplaque CD31 (Figures 1A–D), VEGFA (Figures 1A,E), and VEGFR2 (Figures 1B,F) expression were examined. We showed that intraplaque CD31 were significantly downregulated and microvessel numbers were reduced in *Sema7A^-/-^ApoE^-/-^* mice compared with *Sema7A^+/+^ApoE^-/-^* littermates (Figures 1C,D). Furthermore, VEGFA and VEGFR2 were downregulated without colocalizing with intraplaque CD31 in the lesions at the aortic root of *Sema7A^-/-^ApoE^-/-^* mice compared with *Sema7A^+/+^ApoE^-/-^*, suggesting that Sema7A deficiency ameliorates intraplaque neovascularization in *ApoE^-/-^* mice.

### Deletion of Sema7A Reduces the Accumulation of T Cells, Macrophages, DCs and Reinforces Plaque Stability in *ApoE^-/-^* Mice

Vascular inflammation and neovascularization collaborate to promote atherosclerosis (Kaiser et al., 1999). For instance, T cells play a crucial role in the development of intraplaque neovascularization. Activated T cells are a known source of angiogenic factors (including VEGF), which stimulate angiogenesis and are closely associated with early recruitment of leukocytes (Hansson, 2001). Macrophages are major cellular components in atherosclerotic plaques (Moore et al., 2013; Randolph, 2014). Sema7A was reported to promote the migration of monocytes *in vitro* (Holmes et al., 2002) and DCs *in vivo* (van Rijn et al., 2016). Therefore, we examined the content of macrophages, CD4^+^ T cells, and CD11c^+^ DCs in the plaques at the aortic root by immunostaining. In *Sema7A^-/-^ApoE^-/-^* mice, the areas positively stained for macrophages (24.7 ± 2.8%), CD4^+^ T cells (14.3 ± 1.9%), and CD11c^+^ DCs (9.4 ± 1.3%) were reduced by 58.4, 51.3, and 60.4%, respectively, compared with those in *Sema7A^+/+^ApoE^-/-^* littermates (macrophages: 59.4 ± 4.3%; CD4^+^ T cells: 29.4 ± 1.6% CD11c^+^ DCs: 23.7 ± 2.1%) (Figures 1G–L). In contrast, positive staining areas for neutrophils and SMCs in the aortic root plaques were similar in *Sema7A^+/+^ApoE^-/-^* and *Sema7A^-/-^ApoE^-/-^* littermates (Supplementary Figure S1). These results support a role of Sema7A in recruiting monocytes, T cells, and DCs to atherosclerotic plaques, thereby promoting intraplaque neovascularization and lesion progression.

The newly formed blood vessels of atherosclerotic plaques are extremely vulnerable because of a thin wall and high bleeding risk that lead to plaque instability (Moreno et al., 2004). To determine the effect of Sema7A on plaque stability, collagen deposition of the aortic root was determined by Masson’s trichrome staining. Results showed that the collagen content in *Sema7A^-/-^ApoE^-/-^* plaques was significantly higher than that in *Sema7A^+/+^ApoE^-/-^* plaques (Figures 1M,N, 89.1 ± 0.8% vs. 79.5 ± 1.3%, *P* < 0.001). The vulnerability index, calculated by macrophage contents, lipid content, collagen content, and smooth muscle cell content, was employed to estimate the stability of atherosclerotic plaques (macrophage content% + lipid content%)/(collagen content% + smooth muscle cell content %). *Sema7A^-/-^ApoE^-/-^* plaques was less vulnerable than those of the *Sema7A^+/+^ApoE^-/-^* plaques (Figure 1O, 0.5 ± 0.1 vs. 0.9 ± 0.1, *P* < 0.0001). Taken together, these results suggest that Sema7A deletion suppresses plaque neovascularization and enhances plaque stability in *ApoE^-/-^* mice.

### Sema7A Promotes Migration and Angiogenesis in HUVECs

**FIGURE 2:**
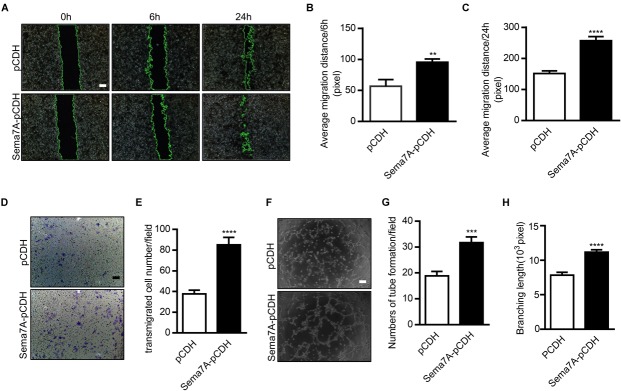
Overexpression of Sema7A promotes endothelial cell migration and tube formation. **(A)** Lenti-pCDH-hSema7A-GFP-transduced HUVECs and Lenti-pCDH-GFP-transduced control HUVECs were cultured and then subjected to wound healing assay at selected time points. Phase contrast images are from the start of the assay (0 h) and after 6/24 h. Location of initial scratch margins indicated by dashed green lines. Bar = 100 μm. **(B,C)** Quantified distances of Sema7A overexpression on cell migration from **(A)** for 6/24 h migration ability. Data are shown as mean ± SEM. Results are representative of ≥ 3 independent experiments. ^∗∗^*P* < 0.01; ^∗∗∗^*P* < 0.001. **(D)** Representative images of transwell membranes with cells stained by crystal violet. Membranes were fixed for 24 h after seeding. Bar = 50 μm. **(E)** The number of migrated cells in Sema7A-pCDH-HUVECs and control pCDH-HUVECs from D. Data are shown as mean ± SEM. Results are representative of ≥ 3 independent experiments. ^∗∗∗∗^*P* < 0.0001. **(F)** Sema7A-pCDH-HUVECs and control pCDH-HUVECs are subjected to *in vitro* tube formation assay. Bar = 50 μm. **(G,H)** Quantified data from F for tube formation numbers **(G)** and total branching length **(H)**. Data are shown as mean ± SEM. Results are representative of ≥ 3 independent experiments. ^∗∗∗^*P* < 0.001; ^∗∗∗∗^*P* < 0.0001.

The reduction of plaque neovascularization by Sema7A deletion suggested a pro-angiogenesis role of Sema7A during atherosclerosis. To elaborate the mechanism by which Sema7A mediates angiogenesis, we first examined the role of Sema7A in endothelial cell migration using a wound scratch assay (0, 6, 24 h) in a Sema7A-overexpressing endothelial cell line (Sema7A-pCDH-HUVECs) versus the control (pCDH-HUVECs). Since the expression of Sema7A retains a low level at baseline (Supplementary Figure S2), we generated a human Sema7A-overexpressing HUVEC line with a GFP tag (Hu et al., 2018). Results showed that Sema7A overexpression significantly enhanced cell migration at 6 h (Figures 2A,B, 6 h, 95.5 ± 5.1 vs. 56.5 ± 11.0, *P <* 0.01) and 24 h (Figures 2A,C, 24 h, 257.1 ± 13.2 vs. 151.6 ± 8.2, *P <* 0.001) after scratching. The effect of Sema7A on the migration of HUVECs was further confirmed in a transwell assay (Figures 2D,E, 85.1 ± 7.2 vs. 37.7 ± 3.7, *P <* 0.0001). We then investigated the pro-angiogenic response of Sema7A by examining the formation of tube-like capillary structures in matrix-gel using Sema7A-overexpressing HUVECs. The ability of capillary tube formation was remarkably strengthened in Sema7A-pCDH-HUVECs compared with pCDH-HUVECs (Figures 2F–H, 31.8 ± 2.2 vs. 18.8 ± 1.8, *P <* 0.001). These results suggest that Sema7A promotes endothelial cell migration and angiogenesis.

### Sema7A Mediates VEGFA/VEGFR2-Promoted Endothelial Cell Migration and Tube Formation in β1 Integrin-Dependent Manner

**FIGURE 3:**
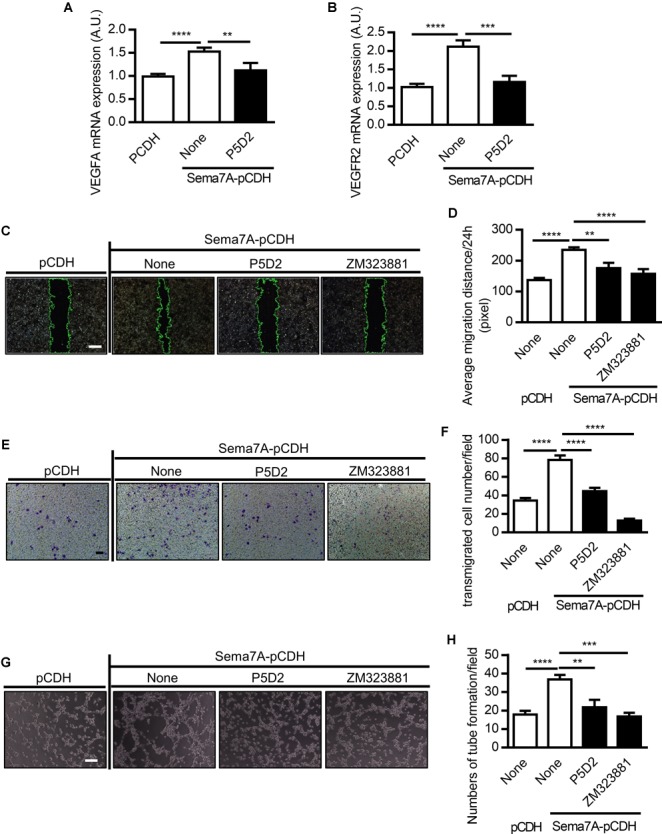
Sema7A enhances VEGFA/VEGFR2 induced cell migration and tube formation through integrin β1. VEGFA **(A)** and VEGFR2 **(B)** mRNA expression in Sema7A-pCDH-HUVECs and pCDH-HUVECs were analyzed by QPCR. Cells were further treated with integrin β1 blocking antibody (P5D2, 1 μg/ml) before VEGFA **(A)** and VEGFR2 **(B)** detected. Data are shown as mean ± SEM. Results are representative of ≥ 3 independent experiments. A.U = arbitrary unit. ^∗∗^*P* < 0.01; ^∗∗∗^*P* < 0.001; ^∗∗∗∗^*P* < 0.0001. **(C)** Sema7A-pCDH-HUVECs and pCDH-HUVECs were treated with integrin β1 blocking antibody (P5D2, 1 μg/mL) or VEGFR2 inhibitor (ZM323881, 10 μM) for 24 h and then subjected to wound healing assay at selected time points. Phase contrast images are from the start of the assay (0 h) and after 24 h. Location of initial scratch margins indicated by dashed green lines. Bar = 100 μm. **(D)** Quantified data from **(C)** for 24 h migration ability. Data are shown as mean ± SEM. Results are representative of ≥ 3 independent experiments. ^∗∗^*P* < 0.01; ^∗∗∗∗^*P* < 0.0001. **(E)** Representative images of transwell membranes with cells stained by crystal violet. Membranes were fixed 24 h after seeding. Cells were prepared as in **(C)**. Bar = 50 μm. **(F)** Quantified numbers of migrated cells in Sema7A-pCDH-HUVECs and control pCDH-HUVECs. Data are shown as mean ± SEM. Results are representative of ≥ 3 independent experiments. ^∗∗∗∗^*P* < 0.0001. **(G)** Sema7A-pCDH-HUVEC and control pCDH-HUVECs were subjected to tube formation assay followed blocking pretreatment as in **(C)**. Bar = 50 μm. **(H)** Quantified data from **(G)** for tube formation numbers. Data are shown as mean ± SEM. Results are representative of ≥ 3 independent experiments. ^∗∗^*P* < 0.01; ^∗∗∗^*P* < 0.001; ^∗∗∗∗^*P* < 0.0001.

VEGF functions as a stimulator of endothelial cell proliferation and migration, resulting in an increase in vascular permeability and apoptosis of endothelial cells (Zachary, 2003). As members of the semaphorin family have been reported to regulate angiogenesis by cooperating with VEGF or modulating VEGF signaling (Segarra et al., 2012; Zhou et al., 2012), we investigated whether Sema7A promotes angiogenesis through VEGFA. We found that both VEGFA and VEGFR2 were dramatically upregulated in Sema7A overexpressing HUVECs (Figures 3A,B). As β1 integrin is a Sema7A receptor that has been shown to mediate cell-cell interaction in axon guidance and T cell response (Pasterkamp et al., 2003; Suzuki et al., 2007), we then determined whether Sema7A enhances VEGFA-mediated angiogenesis through β1 integrin using the β1 integrin blocking antibody P5D2. Results showed that blockage of β1 integrin significantly reduced VEGFA and VEGFR2 mRNA expression (Figures 3A,B).

To examine the functional role of β1 integrin and VEGFA/VEGFR2 in Sema7A induced angiogenesis, the monolayers of HUVECs were incubated in the presence of P5D2 and ZM323881 for 24 h following scratch and cell migration to the predefined area was examined. P5D2 and ZM323881 pre-treatment significantly reduced the migration ability of endothelial cells compared with that of the non-treatment controls (Figures 3C,D). Consistent results were obtained in the transwell assay (Figures 3E,F). Moreover, we examined the effect of β1 integrin and VEGFR2 on tube formation, which reflects the later stages of the angiogenesis. Results showed that Integrin β1 and VEGFR2 blockage significantly reduced the formation of tube-like structures (Figures 3G,H).

### Overexpression of Sema7A Enhances FAK-MAPK Signaling

**FIGURE 4:**
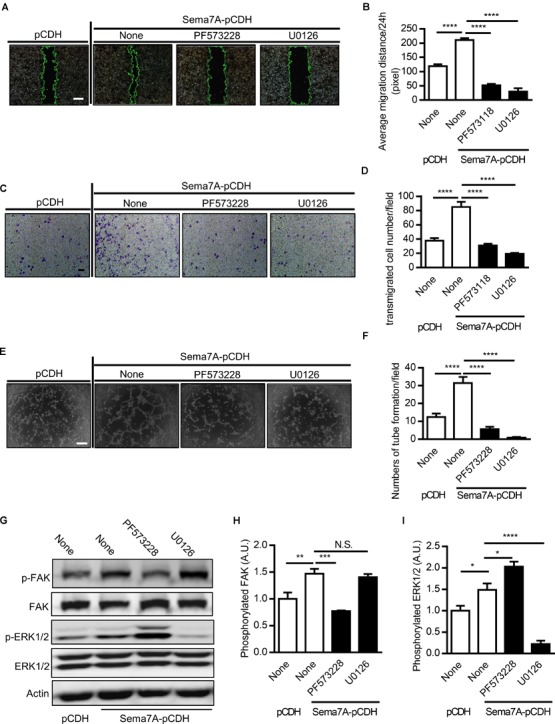
Sema7A upregulates endothelial cell migration and tube formation through FAK/MAPK signaling pathway. **(A)** Sema7A-pCDH-HUVECs and pCDH-HUVECs were treated with FAK inhibitor (PF573228, 10 μM) or MAPK inhibitor (U0126, 20 μM) for 24 h and then subjected to wound healing assay at selected time points. Phase contrast images are from the start of the assay (0 h) and after 24 h. Location of initial scratch margins indicated by dashed green lines. Bar = 100 μm. **(B)** Quantified migration distances from A for 24 h migration ability. Data are shown as mean ± SEM. Results are representative of ≥ 3 independent experiments. ^∗∗∗∗^*P* < 0.0001. **(C)** Representative images of transwell membranes with cells stained by crystal violet. Cells were prepared as in **(A)**. Bar = 50 μm. ***(D)** Quantified numbers of migrated cells in Sema7A-pCDH-HUVECs and control pCDH-HUVECs. Data are shown as mean ± SEM. Results are representative of ≥ 3 independent experiments. ^∗∗∗∗^*P* < 0.0001. **(E)** Sema7A-pCDH-HUVECs and control pCDH-HUVECs subjected to tube formation assay after pretreatment same as in **(A)**. Bar = 50 μm. **(F)** Quantified data from **(E)** for tube formation numbers. Data are shown as mean ± SEM. Results are representative of ≥ 3 independent experiments. ^∗∗∗∗^*P* < 0.0001. **(G–I)** P-FAK **(G,H)**, P-ERK1/2 proteins **(G,I)** were analyzed by western blotting normalized to total FAK/ERK1/2 and displayed as fold-changes relative to control pCDH-HUVECs. Sema7A-pCDH-HUVECs and control pCDH-HUVECs were pretreated same as in **(A)**. Data are shown as mean ± SEM. Results are representative of ≥ 3 independent experiments. A.U = arbitrary unit. ^∗^*P* < 0.05; ^∗∗^*P* < 0.01; ^∗∗∗^P < 0.001; ^∗∗∗∗^*P* < 0.0001.*

Focal adhesion kinase (FAK) is a cytoplasmic tyrosine kinase that plays an essential role in integrin-mediated signal transduction. Upon the combination of VEGFA and VEGFR2, FAK and PI3K signaling pathways are activated to promote migration of endothelial cells (Qi and Claesson-Welsh, 2001). Studies showed that by binding to receptor β1 integrin, Sema7A activates the downstream FAK and MAPK signaling pathways (Khurana et al., 2005). Furthermore, ERK1/2, one of the major targets of the MAPK signaling pathway, are involved in the regulation of angiogenesis in different aspects, including cell proliferation, migration, and survival (Risau, 1997; Pages et al., 2000). To examine integrin-mediated signal transductions in Sema7A-induced angiogenesis, we treated Sema7A-pCDH-HUVECs with the FAK inhibitor (PF573228) and MAPK inhibitor (U0126) for 24h and performed wound healing (Figures 4A,B), transwell (Figures 4C,D), and tube formation assays (Figures 4E,F). Both inhibitors reduced the number and mobility of migrated cells (Figures 4A–D) and tube formation (Figures 4E,F) compared with the vehicle control. Further studies showed that Sema7A-pCDH-HUVECs exhibited higher phosphorylated FAK and ERK1/2 levels than pCDH-HUVECs, which were blocked by the treatment with aforementioned inhibitors (Figures 4G–I). These results suggest that vascular Sema7A upregulation activates FAK, ERK1/2, and regulates the formation of blood vessels.

### Sema7A Promotes ROCK-MYPT-Induced Actin Polymerization, Leading to Cytoskeleton Contraction and Remodeling

**FIGURE 5:**
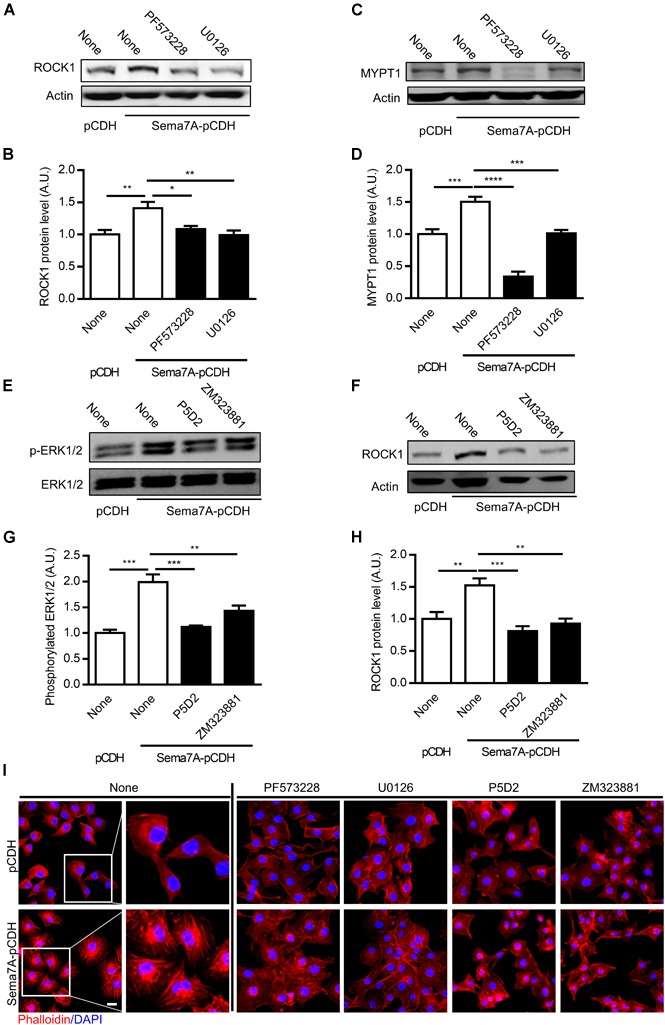
ROCK1 and MYPT1 are involved in Sema7A induced endothelial cell migration and tube formation through FAK/MAPK signaling pathway in a β1 integrin-dependent manner. ROCK1 **(A,B)** and MYPT1 **(C,D)** proteins were analyzed by western blotting normalized to β actin and displayed as fold-changes relative to pCDH-HUVECs. Sema7A pCDH-HUVECs and pCDH-HUVECs were treated with FAK inhibitor (PF573228, 10 μM) or MAPK inhibitor (U0126, 20 μM) for 24 h. Data are shown as mean ± SEM. Results are representative of ≥ 3 independent experiments. ^∗^*P* < 0.05; ^∗∗^*P* < 0.01; ^∗∗∗^*P* < 0.001; ^∗∗∗∗^*P* < 0.0001. **(E,F)** P-ERK1/2 proteins were analyzed by western blotting normalized to total FAK/ERK1/2 and displayed as fold-changes relative to control pCDH-HUVECs. **(G,H)** ROCK1 proteins were analyzed by western blotting normalized to β actin and displayed as fold-changes relative to pCDH-HUVECs. Sema7A-pCDH-HUVECs and pCDH-HUVECs were treated with integrin β1 blocking antibody (P5D2, 1 μg/mL) or VEGFR2 inhibitor (ZM323881, 10 μM) for 24 h. Data are shown as mean ± SEM. Results are representative of ≥ 3 independent experiments. A.U = arbitrary unit. ^∗∗^*P* < 0.01; ^∗∗∗^*P* < 0.001; ^∗∗∗∗^*P* < 0.0001. **(I)** Sema7A-pCDH-HUVEC and control pCDH-HUVECs cytoskeleton were stained by Phalloidin following pretreatment with blocking antibody or inhibitors as shown in figures. Bar = 10 μm. Results are representative of ≥ 3 independent experiments.

Actin reorganization, an important process in VEGF-induced cell migration and vascularization (Gong et al., 2004), is regulated by MAPK (Aplin and Juliano, 1999; Kayyali et al., 2002) and FAK (Kobayashi-Sakamoto et al., 2010) activation. ROCK (Rho-associated coiled forming protein kinase), the downstream kinase of FAK/MAPK signaling pathway, is a major regulator of the actomyosin cytoskeleton which promotes contractile force generation and actin cytoskeleton organization (Riento and Ridley, 2003). MYPT (myosin phosphatase targeting subunit), one of the subunits of myosin phosphatase, regulates the interaction of actin and myosin in response to signaling through the small GTPase Rho and ROCK. Whether ROCK and MYPT are required for Sema7A-integrin β1 promoted VEGFA/VEGFR2-mediated cell migration remains unknown. Using Sema7A-pCDH-HUVECs, we found that ROCK1 (Figures 5A,B) and MYPT1 (Figures 5C,D) were upregulated by Sema7A overexpression, which was blocked by suppression of FAK/MAPK pathway, suggesting a pivotal role of ROCK1 and MYPT1 in Sema7A induced migration and angiogenesis.

Since Sema7A/β1 integrin and VEGFA/VEGFR2 were showed as upstream of FAK/MAPK signaling pathway and actin cytoskeleton organization, we determined whether ERK1/2 and ROCK1 is regulated through β1 integrin/VEGFR2 using an anti-β1 integrin blocking antibody P5D2 and VEGFR2 inhibitor ZM323881, respectively. Results showed that blockage of β1 integrin and VEGFR2 inhibition significantly reduced ERK1/2 activation (Figures 5E,F) and ROCK1 expression (Figures 5G,H). To determine the role of Sema7A in actin polymerization and remodeling of endothelial cells, HUVECs cytoskeleton was stained by phalloidin. Results showed that actin polymerization and stress fiber formation were dramatically reinforced in Sema7A-overexpressing HUVECs compared with pCDH controls (Figure 5I). To ask whether Sema7A-integrin β1 interaction induces actin polymerization through FAK/MAPK signaling pathway, FAK inhibitor (PF573228), MAPK inhibitor (U0126), VEGFR2 inhibitor (ZM323881), and integrin β1 blocking antibody (P5D2) were added to Sema7A-pCDH-HUVECs and pCDH-HUVECs for 24 h followed by phalloidin staining. Incubation with those inhibitors showed depolymerized actin and suppressed stress fiber formation, implying that Sema7A binding to integrin β1 regulates cytoskeleton remodeling via FAK/MAPK signaling pathways in HUVECs (Figure 5I).

## Discussion

In this study, we investigated the role of Sema7A in angiogenesis and atherosclerotic neovascularization. Using Sema7A-overexpressing HUVECs, we showed that Sema7A promotes VEGFA/VEGFR2-mediated cell migration and angiogenesis by activating β1 integrin and its downstream signaling molecules FAK and ERK1/2, as well as ROCK1 and downstream MYPT1 that are responsible for actin polymerization. *In vivo* studies indicated that deletion of Sema7A reduces VEGFA/VEGFR2 expression, neovascularization, decreases the recruitment of T cells, macrophages, and DCs in the atherosclerotic plaques, and reinforces plaque stability.

Previous reports showed that Sema7A induces neovascularization in corneal fibroblasts and murine mammary tumor cells (Ghanem et al., 2011; Garcia-Areas et al., 2014). As for corneal angiogenesis, the study revealed that Sema7A expressed in bFGF (basic fibroblast growth factor) stimulated fibroblasts and mediates vascular growth by stimulated fibroblasts (Ghanem et al., 2011). In the tumor study, Sema7A was highly expressed in murine mammary tumor cells and peritoneal elicited macrophages. Sema7A in mammary carcinomas induces macrophages to secret chemokines such as CXCL2/MIP-2 to support angiogenesis in the tumors (Garcia-Areas et al., 2014) However, the cellular basis of Sema7A receptors and the molecular mechanisms were not fully described. In this study, we showed endothelial Sema7A promotes angiogenesis and intimal neovascularization via upregulating endothelial cell mobility and cytoskeleton reorganization. The molecular investigation indicates that Sema7A promotes VEGFA/VEGFR2-mediated cell migration and angiogenesis by activating β1 integrin and its downstream FAK and ERK1/2 signaling pathways. This finding extends our understanding of the role of Sema7A in angiogenesis and atherosclerosis (Hu et al., 2018).

Endothelial cells regulate endothelial barrier function, which control the efflux of plasma proteins and infiltration of blood cell into the subendothelium (Wallez and Huber, 2008). Incomplete endothelium structure leads to increased vascular permeability, which is inseparable from chronic inflammation and tumor angiogenesis (Wallez and Huber, 2008; Dejana et al., 2008, 2009). VEGF was initially described as an endothelial cell-specific mitogen and an effective angiogenic factor (Ferrara, 1999) and plays a crucial role in facilitating intimal neovascularization (Celletti et al., 2001). Consistently, our investigation indicated that Sema7A overexpression increases the expression of VEGFA and VEGFR2, and shifts endothelial cells to a pro-angiogenic phenotype. VEGF signaling pathways depend on the interaction between VEGFRs and integrins (Weis and Cheresh, 2005). VEGFRs take a crucial part in intercellular adhesion through direct interaction with intercellular adhesion molecules. In endothelial cells, VEGFR2 binds to vascular endothelial (VE) cadherin, a major protein responsible for maintaining endothelial barrier integrity, regulating the integrity of the junction. In contrast, the microvessels located in the atherosclerotic plaques of the progressive stage showed damage to the morphology and integrity of endothelial cells and loss of VE-cadherin (Bobryshev et al., 1999; Sluimer et al., 2009). To explore the relationship between endothelial Sema7A and defects in endothelial permeability, we measured VE-cadherin expression in Sema7A-overexpressing HUVECs, which is thought to be released once endothelium integrity damaged and extensively stained in damaged endothelium (Boneu et al., 1975). In comparison with pCDH controls, Sema7A overexpressing HUVECs showed a significantly less continuous formation of VE-cadherin coverage (Supplementary Figure S3). These results suggest that endothelial Sema7A is associated with areas of intraplaque angiogenesis potentially containing defective endothelial barrier integrity and function.

The causal role of Sema7A in experimental atherosclerosis urges a translational outlook into its clinical relevance. To date, increased systemic or tissue levels of Sema7A have been associated with a spectrum of inflammatory disorders, including multiple sclerosis (Costa et al., 2015), interstitial lung disease (Gan et al., 2011), rheumatoid arthritis (Xie and Wang, 2017), and airway allergy (Esnault et al., 2014). In our preliminary study, we found that elevated serum Sema7A level in patients with acute ischemic stroke compared with healthy controls (unpublished data). The augmentation of Sema7A may be attributed to injured endothelium and activated blood cells, as well as infiltrated leukocytes inside the ruptured plaque. Again, whether Sema7A is also increased in stable atherosclerotic diseases remains uncertain. To establish the essential role of Sema7A as a potential diagnostic marker, future works are required to determine its reference range in circulation with a larger and stratified population. Examples of these efforts may include the studies with prospective design to explore the prognostic value of Sema7A for major cardiovascular outcomes and targeting Sema7A to provide a novel avenue in combating cardio-cerebrovascular diseases. Designing antagonizing peptides, screening for potential small molecule agents, and developing monoclonal antibodies to inhibit Sema7A are currently under progression.

In this study, we investigated the role of Sema7A underlying angiogenesis in atherosclerosis using a knockout mice model and EC Sema7A expression system. Our results demonstrated that Sema7A enhances cell migration and capillary network formation in endothelial cells. Moreover, Sema7A deletion significantly attenuates inflammatory cell infiltration and neovascularization in the plaque of atherosclerotic mice. Although the differential contribution of cells to neovascularization in the plaques and plaque stability require further evaluation, our results suggest that targeting Sema7A as a potential method to control angiogenesis for cardiovascular medicine.

## Ethics Statement

This study was carried out in accordance with the recommendations of the Institutional Animal Care and Use Committee of Soochow University. The protocol was approved by the Institutional Animal Care and Use Committee of Soochow University.

## Author Contributions

SH and YL contributed equally to this article as first authors. SH, YL, and LZ contributed conception and design of the study. SH and YL organized the database and performed the statistical analysis. YL wrote the first draft of the manuscript. SH, TY, and LZ wrote sections of the manuscript. All authors contributed to manuscript revision and approved the submitted version.

## Conflict of Interest Statement

The authors declare that the research was conducted in the absence of any commercial or financial relationships that could be construed as a potential conflict of interest.
