# Supplementary figures and images for: Semaphorin 7A Promotes VEGFA/VEGFR2-Mediated Angiogenesis and Intraplaque Neovascularization in ApoE-/- Mice
Source: Front Physiol. 2018 Nov 30;9:1718. doi: 10.3389/fphys.2018.01718 (PMC6284023; doi:10.3389/fphys.2018.01718)

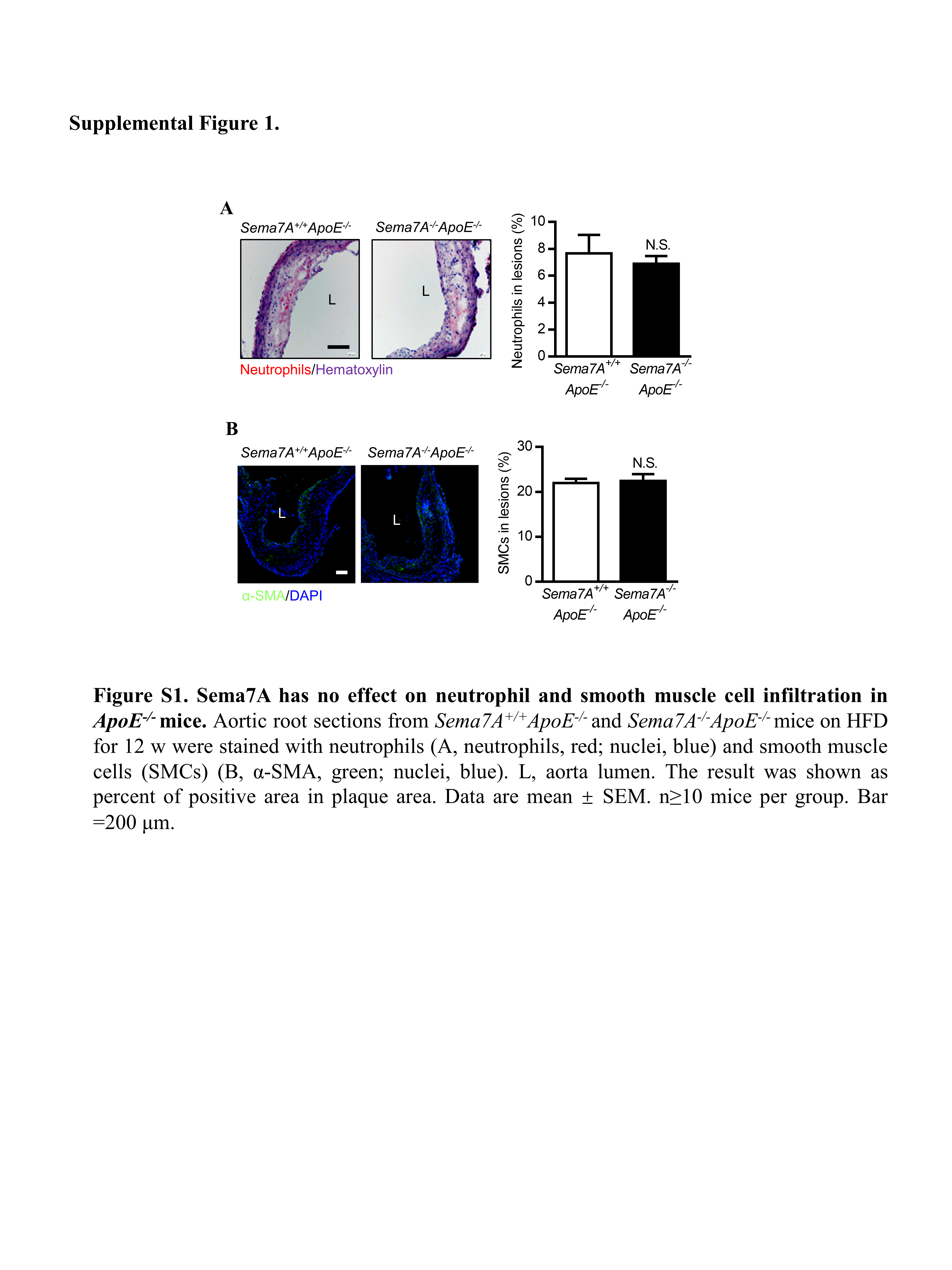

Supplement: Supplementary file 1 [file Image_1.tif]

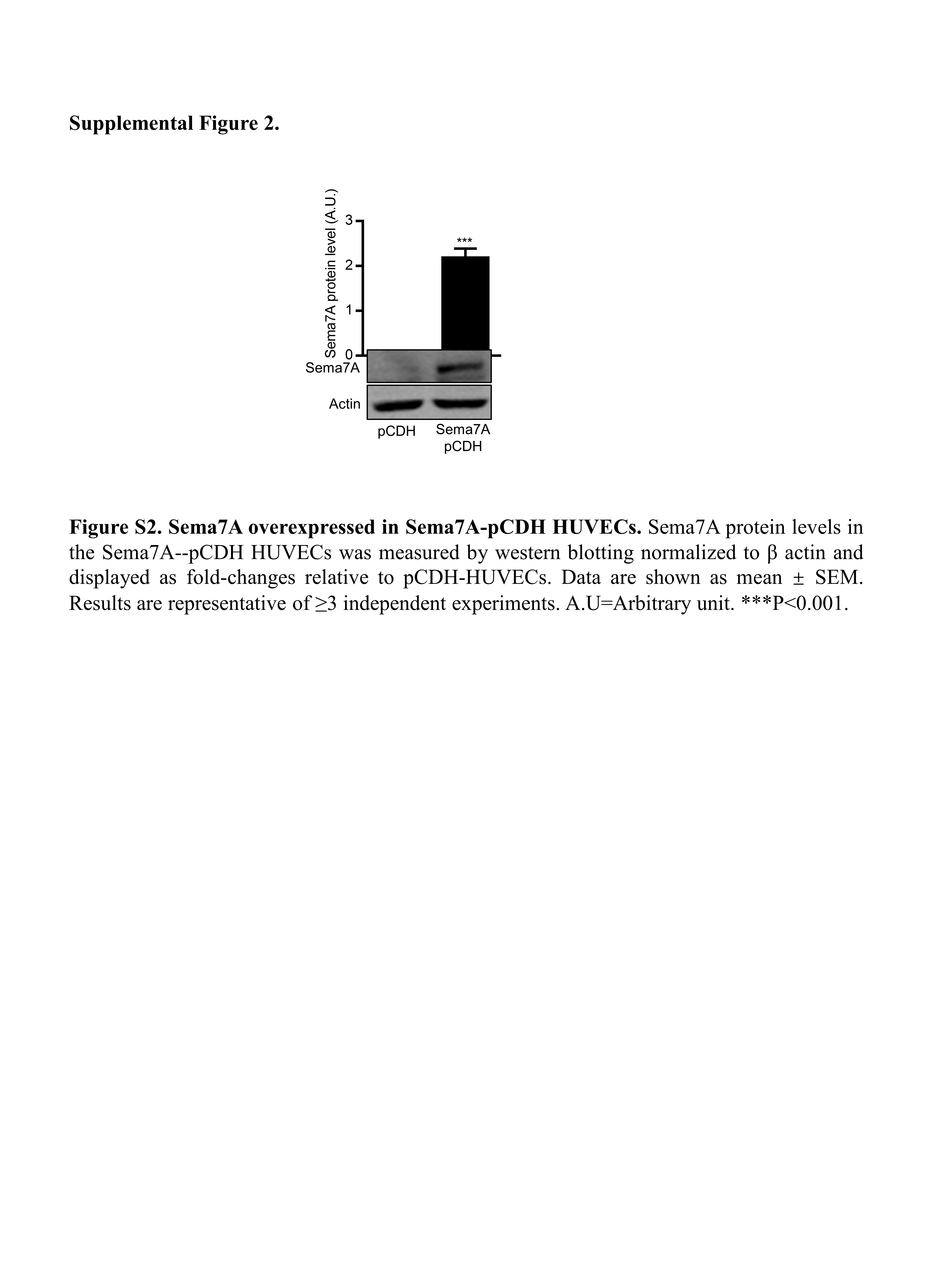

Supplement: Supplementary file 2 [file Image_2.tif]

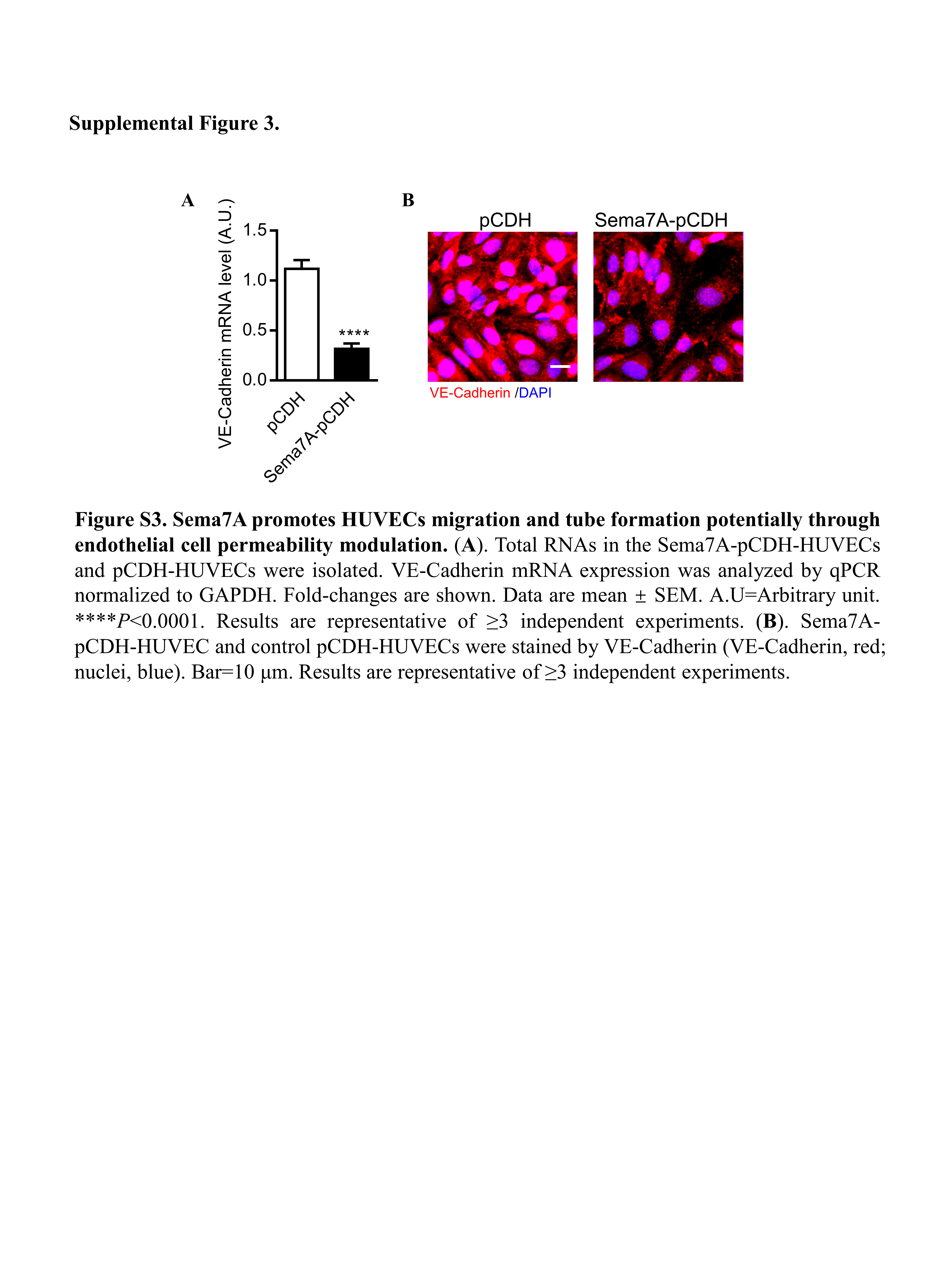

Supplement: Supplementary file 3 [file Image_3.tif]
