# Supplementary material for: Semaphorin 7A Promotes VEGFA/VEGFR2-Mediated Angiogenesis and Intraplaque Neovascularization in ApoE-/- Mice
Source: Front Physiol. 2018 Nov 30;9:1718. doi: 10.3389/fphys.2018.01718 (PMC6284023; doi:10.3389/fphys.2018.01718)
Supplement: Supplementary file 4 [file Table_1.DOCX]

| **Gene** | **Forward (5’---3’)** | **Reverse (5’---3’)** |
| --- | --- | --- |
| VEGFA (human) | AGGGCAGAATCATCACGAAGT | AGGGTCTCGATTGGATGGCA |
| VEGFR2(human) | TCGGAAATGACACTGGAGCC | CCCGAGACATGGAATCACCA |
| VECadherin (human) | GTGAGTCGCAAGAATGCCAA | GAACAACCGATGCGTGAACA |
| GAPDH (human) | GAAAGCCTGCCGGTGACTAA | GCATCACCCGGAGGAGAAAT |

Supplemental Table 1. Primers used for qPCR
